# Caries preventing efficacy of new Isomalt-containing mouthrinse formulations: a microbial study

**DOI:** 10.1038/s41405-024-00241-4

**Published:** 2024-06-18

**Authors:** Bennett T. Amaechi, Parveez Ahamed AbdulAzees, Sahar Mohseni, Minh N. Luong, Chun-Yen Lin, Maria Camila Restrepo-Ceron, Yuko Kataoka, Temitope O. Omosebi, Kannan Kanthaiah

**Affiliations:** 1 Department of Comprehensive Dentistry, University of Texas Health, San Antonio, TX USA; 2 Department of Dentistry, Tri-Service General Hospital, Taipei City, Taiwan, ROC; 3 CES University, Medellín, Colombia; 4 Department of Restorative Dentistry, Lagos State University Teaching Hospital, Ikeja, Nigeria

**Keywords:** Health care, Preventive dentistry

## Abstract

**Objectives:**

The effectiveness of an Isomalt-containing mouthrinse to prevent caries development was investigated.

**Methods:**

Human enamel blocks were randomly assigned to five groups (*n* = 30/group): De-ionized distilled water (DDW), and mouthrinse containing either (IFC) 1% Isomalt, 225 ppm fluoride, and 0.05% cetylpyridinium chloride (CPC), (IF) 1% Isomalt and 225ppm fluoride, (FC) 225 ppm fluoride and 0.05% CPC or (F) 225 ppm fluoride. During 7-day demineralization in a Microbial Caries Model, mouthrinses were applied once daily for 1 min. Demineralization was assessed using Surface Microhardness testing for percentage change in SMH (%ΔSMH) and Transverse Microradiography for mineral loss (ΔZ). Data analysis (α = 0.05) used paired t-test (Intra-group comparison using SMH) and ANOVA/Tukey’s for inter-group comparisons (%ΔSMH and ΔZ).

**Results:**

With SMH, relative to sound enamel baseline, demineralization was significant (*P* < 0.001) in all groups, except in IFC. Intergroup comparison with %ΔSMH showed significantly (*p* < 0.001) greater demineralization in DDW compared to other groups, and in IF, FC, and F compared to IFC (*P* < 0.001). With ΔZ, relative to DDW, all groups significantly (*p* < 0.0001) inhibited demineralization at varying percentages.

**Conclusions:**

Mouthrinse containing Isomalt, fluoride, and CPC inhibited demineralization amidst cariogenic biofilm; thus, highlighting its potential as a more effective caries control tool than mouthrinse with only fluoride.

## Introduction

Caries remains one of the most widespread multifactorial diseases in the world caused by the dynamic process of demineralization and remineralization at the interface between biofilm and the tooth surface [1, 2]. Dental caries develops as a consequence of interaction between different etiological factors such as cariogenic microorganisms and frequent consumption of fermentable carbohydrates amid poor oral hygiene [3]. Following the metabolism of sugar, cariogenic microorganisms produce organic acids (e.g., lactic acid) that demineralizes tooth tissue leading to manifestation of caries lesion [4]. With large amount of supporting evidence, fluoride is considered the most effective agent in inhibiting tooth demineralization and decreasing the progression of existing lesions [5, 6]. However, the anticaries effect of fluoride in high caries risk situation due to poor oral hygiene is limited since high concentrations of fluoride is required to effectively reduce acid production by bacteria, and such concentrations are not allowed in mouthrinse tailored for frequent application [7]. For this reason, there is need for other strategies that could work synergistically with fluoride to enhance its effectiveness to control dental caries [8, 9]. Moreover, it has become clear that in complex multispecies biofilms polymicrobial interactions enhance the resistance to antimicrobials, thus increasing the resistance to antimicrobial treatment by biofilm-bound bacteria than their planktonic counterparts in saliva [10]. Therefore, it is now acknowledged that studying new compounds aimed at interfering with bacteria activities requires polymicrobial biofilm models instead of traditional bacterial cell cultures [11–13].

The Sugar alcohols, otherwise known as Polyols, are hydrogen-enriched carbohydrates, which are not readily metabolized by bacteria, and as such, are less cariogenic and widely used to replace fermentable sugars in the foods products. Among these sugar alcohols is Isomalt, a hexanopyranosyl-hexitol which is approved by the US Food and Drug Administrations (FDA) as an anticaries agent [14]. There is strong evidence that sugar alcohol suppresses dental caries via inhibition of glucosyltransferases in cariogenic bacteria as well as enhance caries remineralization [15, 16]. Cetylpyridinium chloride (CPC), which carries a long history of safe and effective oral use, has frequently been employed as an antimicrobial ingredient to improve clinical efficacy of oral care products [17, 18].

Based on the above discussion, it is conceivable that combination of Isomalt, fluoride and CPC in a mouthrinse formulation tailored to prevent dental caries could enhance the caries preventive effect of fluoride in such formulation. Therefore, the main objective of the present study was to use a multispecies microbial caries model to investigate the effectiveness of mouthrinse formulations containing Isomalt, fluoride and CPC to prevent tooth surface demineralization, comparing it with mouthrinse containing only fluoride.

## Materials and methods

### Sample preparation

Extracted sound human molars were collected from various clinics of our school of dentistry (SOD) after Institutional Review Board approval (Approval #: HSC20080233N) for collecting unidentifiable extracted teeth. The teeth were sterilized as recommended by the university, brushed with pumice slurry and electric toothbrush (Braun Oral-B Plaque Remover, Proctor & Gamble, Cincinnati, OH, USA), and then examined for absence of malformations. Then square tooth blocks (3 mm length × 3 mm width) of 2 mm thickness were cut from the smooth surfaces of the coronal portion of each tooth using water-cooled diamond wafering blade (Allied High Tech, USA). Using adhesive-back lap-ping film (30–1 µm) in a MultiPrep™ Precision Polishing machine (Allied High Tech, USA), the tooth blocks (150) had their enamel and dentin surfaces polished to achieve flat, plane and parallel surfaces required for surface microhardness (SMH) measurement. After this, the surfaces of the blocks were coated with two layers of acid-resistant nail varnish, except on the enamel surface.

### Measurement of baseline SMH

Using a Knoop diamond indenter (Tukon 2100; Wilson-Instron, Norwood, MA, USA), the baseline SMH (SMH_b_) of each tooth block was measured with 5-s application of a load of 50 g, by placing three indentations spaced by at least by 100 µm on the enamel surface. The software automatically calculated the Knoop hardness numbers and averaged it for each block.

### Experimental procedure

Following SMH_b_ measurement, each of the selected 150 blocks were randomly assigned to one of the following 5 experimental groups (*n* = 30) as shown in Table 1. Allocation of the enamel blocks was based on their SMH_b_ values such that the values of the mean SMH_b_ for the five groups did not differ significantly. Following grouping, the 5 groups were subjected to demineralization by plaque growth in a Microbial Caries Model (MCM) described below.

**Table 1:** Experimental groups and the mouthrinse formulations. DDW (Distilled Deionized water), IFC (Isomalt-fluoride-CPC mouthrinse), IF (Isomalt-fluoride mouthrinse), FC (Fluoride-CPC mouthrinse), F (Fluoride-only mouthrinse).

Mouthrinse #	Distilled Deionized water (DDW)	Isomalt-fluoride-CPC (IFC)	Isomalt-fluoride (IF)	Fluoride-CPC (FC)	Fluoride-only (F)
**Fluoride**	-	225 ppm	225 ppm	225 ppm	225 ppm
**Isomalt**	-	1%	1%	-	-
**CPC**	-	0.05%	-	0.05%	-

The experiment was carried out using our MCM, a multiple-chamber continuous flow bacteria culture system that has been validated and described in our previous studies [11–13]. Each treatment group was assigned to a separate chamber, and the tooth blocks were embedded within a cylindrical acrylic rod inside the chamber, ensuring that the surfaces of the blocks flush with the acrylic surface to permit streamline flow of the culture media on enamel surface to enable the growth of dental plaque on the enamel surface. During operation, the growth media (Todd Hewitt Broth), which simulates the oral fluid (saliva), was circulated continuously through each chamber. Daily meals were simulated by supply of 10% sucrose three times daily for 6 min on each occasion, and this maintained the plaque growth as well as established a pH cycling (demineralization-remineralization episodes). At non-feeding times, the plaque pH in each chamber was monitored. Plaque growth and caries development on the surfaces of the tooth blocks were initiated by 12-h circulation of Todd Hewitt broth inoculated with a mixture of Streptococcus mutans (NCTC 10449, ATCC, Manassas, VA) and Lactobacilli casei (NCIB 8820, ATCC, Manassas, VA) culture (broth to inoculum ratio 10:1) through the chambers on day 1 for the adhesion phase of plaque formation. For the remaining 12 h of day 1, broth without bacteria was circulated. From day 2, the surface of the blocks, which are now covered by plaque, were treated as follows (Table 2). While the negative control group was treated with de-ionized distilled water (DDW), the test groups were treated with their respective mouthrinse formulations, morning and evening, for 2 min on each occasion as follows. The tooth blocks born on the acrylic rods were inserted into 150 ml of the product (DDW or mouthrinse) for 2 min and then gently rinsed in a sterile Phosphate Buffer Saline (PBS). The entire MCM was housed inside a reach-in incubator at 37 °C, and all treatments were carried out under aseptic condition inside the incubator for 7 days.

**Table 2:** Treatment schedule for microbial caries model for this study

Day	Time	Treatment
Day 1	8:00	Circulation of bacteria-free Todd Hewitt Broth (THB) starts.
	10:00–11:00	Bacteria-inoculated THB is circulated for 12 h (adhesion phase)
	11:00	Circulation of bacteria-free THB re-starts.
	20:00	Sucrose circulation for 6 minutes
	20:06 till next morning	Circulation of bacteria-free THB re-starts.
Day 2 – Day 7	7:00	Mouthrinse (1 min) treatment.
	7:02	Circulation of bacteria-free THB re-starts.
	8:00	Sucrose circulation for 6 min
	8:06	Circulation of bacteria-free THB re-starts.
	14:00	Sucrose circulation for 6 min
	14:06	Circulation of bacteria-free THB re-starts.
	19:00	Mouthrinse (1 min) treatment.
	19:02	Circulation of bacteria-free THB re-starts.
	20:00	Sucrose circulation for 6 min
	20:06	Circulation of bacteria-free THB re-starts.

### Post-treatment surface microhardness measurement

On termination of the experiment, the tooth blocks were harvested and processed for demineralization assessment by measuring the post-treatment Surface Microhardness (SMH_t_). The SMH_T_ measurement were performed as described above by three indentations on the free (un-indented) surface of the block, and the average value calculated for each block. At this point the pre-test (SMH_b_) and post-test (SMH_t_) surface microhardness value of the lesions were available. The mean (*n* = 30) values of the SMH_b_ and SMH_T_ was calculated for each treatment group for intragroup comparison. However, to make comparisons between the 5 groups (plus the negative control) or the four mouthwash groups (intergroup comparison), percentage change in SMH (%ΔSMH), calculated relative to the baseline (SMH_b_), was determined for each test product (%ΔSMH was used for intergroup comparison to make provision for the fact that the enamel blocks came from different teeth and as such their baseline SMH may differ). This is calculated thus: % change in SMH (%ΔSMH) = ((SMH_b_ – SMH_t_)/SMH_b_)*100. From this equation, the mean values (± standard deviations) of the %ΔSMH for each of the 5 groups were generated for statistical analysis.

### Post-treatment transverse microradiography and image analysis

Following SMH_T_ measurement, enamel slice of 150 µm thickness was sectioned out of each tooth block by cutting perpendicular to enamel surface of the block using a water-cooled wafering blade (Allied High Tech, USA). Each slice was machine-polished at both sides down to 100 µm thick to obtain planoparallel surfaces for TMR. Then the slices were microradiographed on X-ray glass plates (Micro chrome Technology, CA, USA) with an X-ray generator system (Panalytical, Amsterdam), by exposing the plates for 10 min at 20 kV and 10 mA before processing in the developer and fixer solutions. Processing consisted of a 5-min development in Kodak HR developer and 15 min fixation in Kodak Rapid-fixer before a final 30-min wash period. Then the plates were viewed under an optical microscope, and via a Sony model XC-75CE CCTV camera, the microradiographic images were captured in a computer with the TMR2006 version 3.0.0.6 image analysis program (Inspektor Research, Amsterdam). Using the TMR program, the captured slab images were analyzed along with data from the image of the step wedge used for calibrating the software, and the integrated mineral loss (vol%. µm) was quantified for each demineralized area on each specimen in accordance with the directions in the TMR program [19]. The program defined ‘integrated mineral loss (∆z)’ as the difference in volume percent of mineral between sound and demineralized tissue integrated over the lesion depth [20]. By this method, ∆z (vol%.µm) was quantified for each caries lesion (demineralization) on each enamel block. The mean values (±SD) of the mineral loss in each experimental group (*n* = 30) was calculated.

### Statistical analysis

Stata 11.0 (StataCorp, College Station, TX) statistical software was used for the statistical analysis, and for all statistical tests, *p* < 0.05 was considered significant. The assumptions of equality of variances was checked using Brown-Forsythe test, while the normality distribution of all variables was assessed using the Shapiro–Wilk test. The mean (*n* = 30) values of the SMH_b_ for all groups were compared using One Way Analysis of Variance **(**ANOVA) to ensure there was no significant difference among the groups before treatment. The mean (*n* = 30) values of the SMH_b_ and SMH_t_ for each product group were compared using paired *t* test to determine if there is any significant change (demineralization) in SMH within each group (Intra-group comparison). Using the mean values of the %ΔSMH and **∆**z of each group, the five groups were compared among themselves using ANOVA followed by Tukey’s multiple comparison test.

## Results

### Demineralization assessment by SMH testing

**Fig. 1:**
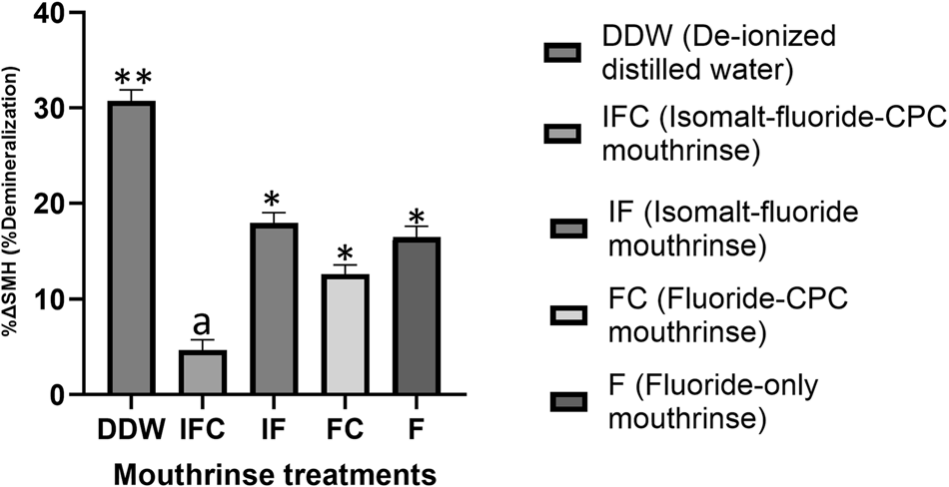
Protection of tooth enamel against demineralization as measured by Surface Microhardness Testing. Mean percentage change in surface microhardness (%∆SMH) after 7 days of treatment with each treatment group. **Significantly greater than all the other groups; ^*^Not significantly different from each other. ^a^Significantly less than IF, FC, & F.

The data passed both the normality test by Shapiro-Wilk (*P* = 0.822) and the equal variance test by Brown-Forsythe (*P* = 0.968). ANOVA indicated there was no significant difference (*p* > 0.05) in the mean values of the baseline surface microhardness (SMH_b_) among the groups prior to test. Following test, paired *t* test showed there was significant demineralization (*P* < 0.001) with DDW, IF, FC, and F, but not with IFC (*p* = 0.303) relative to sound enamel baseline. Comparing the product groups using mean values of %ΔSMH, ANOVA showed a statistically significant difference (*P* < 0.001) among the groups. With Tukey’s test, there was a significantly (*p* < 0.001) greater demineralization with DDW compared to all other treatment groups (Fig. 1). Relative to DDW, all mouthrinse formulations inhibited demineralization at varying percentages (Table 3). There was a significantly greater demineralization (*P* < 0.001) with IF, FC, and F compared to IFC (Fig. 1). There was no statistically significant difference (*p* > 0.05) between these three mouthrinses (IF, FC, and F) formulations.

**Table 3:** Mean (±SD) values of %∆SMH and mineral loss in each experimental group and percentage reduction of demineralization by individual mouthrinse formulation relative to the control group (DDW).

Treatment Groups	%∆SMH (% Demineralization)	% Reduction in demineralization relative to control (DDW)	Mean mineral loss (± SD)	% Reduction in mineral loss relative to control (DDW)
Distilled Deionized water (DDW)	30.8 ± 1.1	-	3059 ± 178	-
Isomalt-fluoride-CPC mouthrinse (IFC)	4.7 ± 1.1	84.8	412 ± 19	87%
Isomalt-fluoride mouthrinse (IF)	17.9 ± 1.1	41.8	2040 ± 107	33%
Fluoride-CPC mouthrinse (FC)	12.6 ± 1.0	59.0	1299 ± 42	58%
Fluoride-only mouthrinse (F)	16.4 ± 1.2	46.6	1915 ± 68	37%

%∆SMH percentage change in surface microhardness; CPC - Cetylpyridinium chloride.

### Demineralization assessment using TMR

**Fig. 2:**
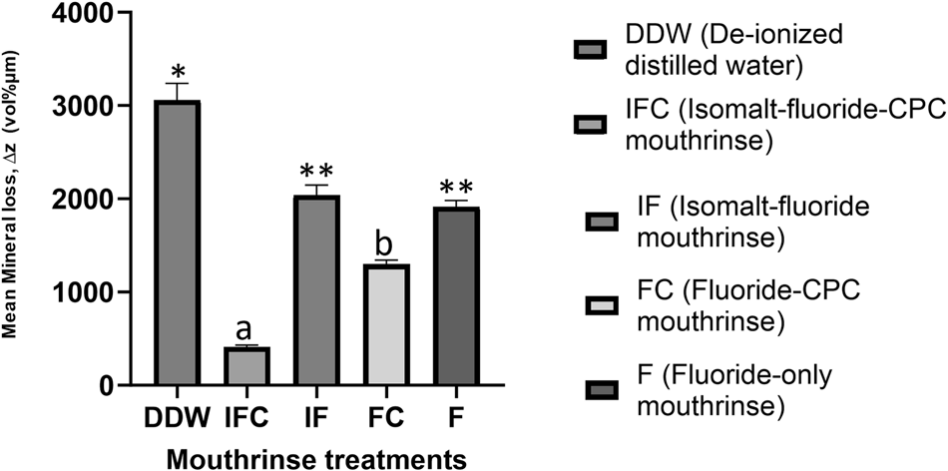
Protection of tooth enamel against demineralization as measured by Microradiography. Mean mineral loss after 7 days of treatment with each treatment group. *Significantly greater than all the other groups; ^a,b^Significantly different from each other; ^**^Not significantly different from each other.

**Fig. 3:**
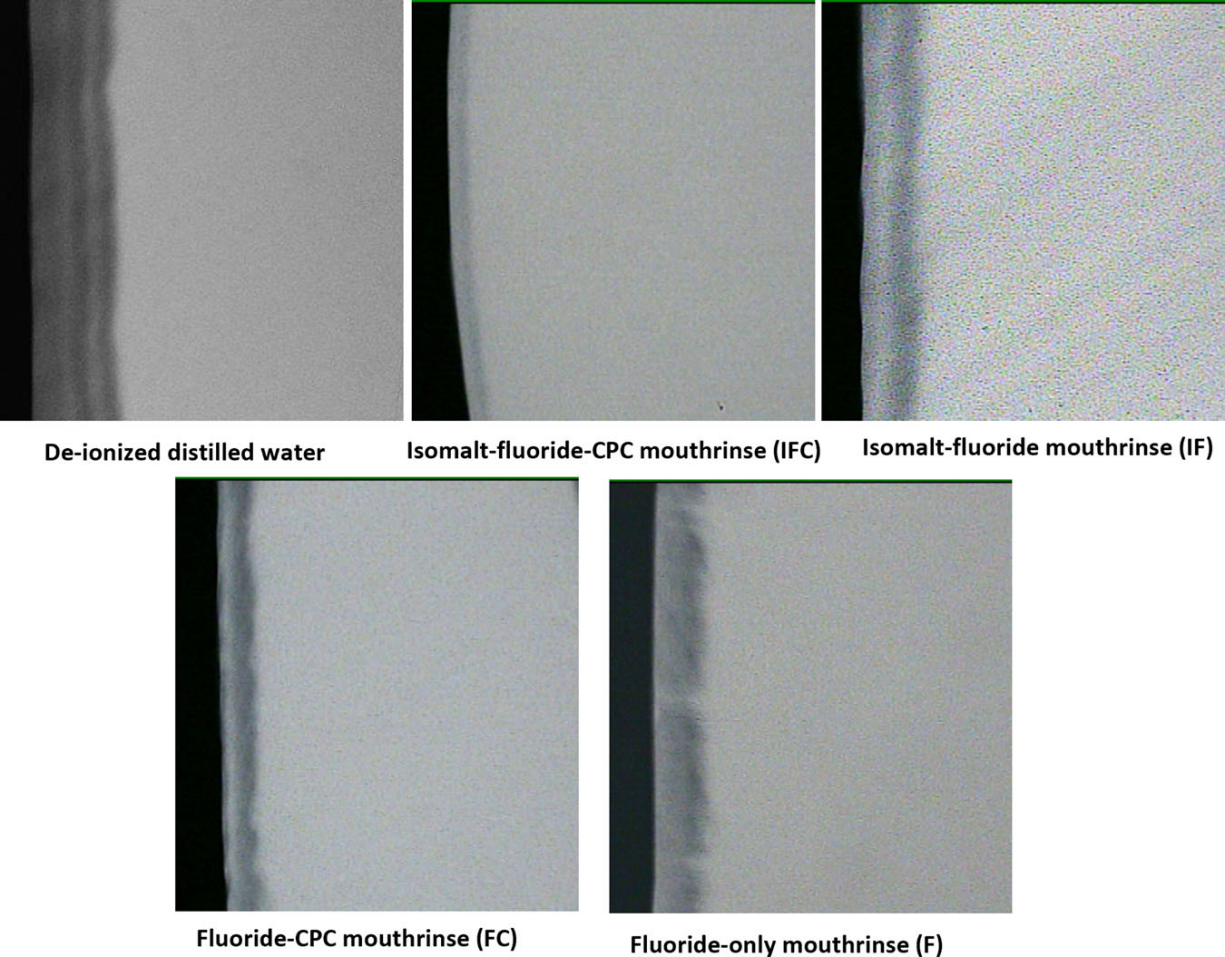
Demineralization observed with each experimental product. Representative microradiographic images from each experimental group depicting the variation in the level of demineralization among the treatment groups after 7 days of treatment.

Intergroup comparison using their mean ∆z, ANOVA indicated that the differences among the groups were statistically significant (*p* < 0.0001), thus the differences among the treatment groups are greater than would be expected by chance. With Tukey’s multiple comparison test, there was a significantly (*p* < 0.001) greater demineralization (Δz) with DDW compared to all treatment groups (Fig. 2). Relative to DDW, all mouthrinse formulations significantly (Tukey’s; *p* < 0.0001) inhibited demineralization (mineral loss) at varying percentages (Table 3). All comparisons of the mouthrinse formulations with each other were statistically significant (Tukey’s; *p* < 0.0001), except for IF vs. F (*p* = 0.9015). Figure 3 showed the representative microradiographic images from each experimental group depicting the variation in the level of demineralization among the treatment groups.

## Discussion

With large amount of supporting evidence, fluoride is considered the most effective agent in inhibiting tooth demineralization and decreasing the progression of existing lesions [6]. The decline in caries prevalence in some parts of the world over the past decades has been attributed to the increasing and routinely use of fluoridated oral hygiene products, particularly toothpaste [6]. However, in high caries risk situation caused by poor oral hygiene, there is a limitation to the effectiveness of fluoride in preventing caries since a high concentration of fluoride is required to effectively reduce acid production by cariogenic bacteria [5]. For this reason, there is need for other strategies that could either work synergistically with fluoride to enhance its effectiveness to control dental caries or serve as a complement to fluoride toothpaste [8, 21]. Considering that most guidelines across the globe recommend the use of mouthwashes as an “adjunct” to good oral hygiene [22, 23], and thus provide a basis for their widespread use to manage key oral diseases, it is conceivable that it can serve as a vehicle for application of any complement tailored to enhance the effectiveness of fluoride in preventing caries. For this reason, the present study investigated the effectiveness of mouthwash formulations that are combining fluoride, Isomalt, and CPC, to prevent tooth surface demineralization, and compared it with formulations without Isomalt and/or CPC. Although most marketed mouthwashes are applied for 30 s, the isomalt mouthwashes investigated in the present study were tailored for 1-min application, thus the 1-minute application used for all the mouthwashes in the present study as directed by the manufacturer. The study was conducted using a multispecies MCM that acts as an artificial mouth, producing cariogenic dental plaque and simulating the biological and physiological activities observed within the oral environment [11–13]. In the present study, the application of the mouthwashes in the presence of plaque, frequently fed with sucrose without toothbrushing, subjected the tooth blocks to the natural demineralization and remineralization cycles similar to a high caries risk condition in the oral environment [12]. The result of the study showed that with either the SMH (%ΔSMH) or the TMR (Δz) measurement, combining fluoride with Isomalt and CPC (IFC) consistently showed higher effectiveness in preventing demineralization (87%) than when it is only fluoride (37%) or fluoride combined with either Isomalt (33%) or CPC (58%) only (Figs. 1 and 2; Table 3). The superior performance of the IFC over the other mouthwash formulations in preventing enamel demineralization was further demonstrated by intragroup comparison of the baseline and post-treatment SMH data, which showed significant demineralization with all other mouthwashes, but not with IFC. This is a demonstration of synergistic effect of these three agents (fluoride, Isomalt, and CPC) in one mouthwash. This synergy is feasible considering that each of these agents has been previously shown to have either antimicrobial effect, noncariogenic or anticaries effects [6, 8, 24, 25], and as such, this effect can be attributed to the agents complementing the effect of each other. Isomalt has been demonstrated in several studies to be non-acidogenic and non-cariogenic [1, 16, 24, 26, 27]. In some of those studies, Isomalt was also reported to have the potential to promote caries preventions as well as remineralization of early caries lesions by binding and concentrating calcium ions in the plaque[16, 24, 26]. Other studies suggest that various sugar alcohols inhibit the growth of S. mutans in the presence of glucose as well as inhibit acid production from glucose by washed cells of S. mutans [28, 29]. Likewise, Cetylpyridinium chloride, a quaternary ammonium compound, has long been used in oral hygiene products, in varying concentrations (0.045%–0.1%), as a safe and effective broad-spectrum antimicrobial agent that reduces plaque and gingivitis [17, 18, 30–35]. In our previous studies, we also demonstrated the caries preventive effect of CPC applied as nanoemulsion [11, 13, 36]. Also, CPC has been shown to impact on the progression and the maturation of the dental plaque by decreasing the size and the connectivity in the bacterial network, especially the gingivitis-related bacteria [37]. It is established that CPCs exhibit their antibacterial action through a reaction with lipids and proteins of the cell membrane, which leads to disorganization in its structure and the leakage of low-molecular components out of the cell [38]. They also release autolytic enzymes leading to the lysis of the bacterial cell wall and loss of functional components. CPC also inhibits fructosyltransferases, an enzyme that synthesizes fructans from sucrose that contributes to development of caries [36]. Furthermore, CPCs exhibit antifungal actions through reverse distribution of charges on the cell surface, and antiviral effects through disruption or detachment of the viral envelope, with subsequent release of the nucleocapsid, but their effects on non-enveloped viruses are less certain [39–41]. Based on the above modes of actions of the three agents in the tested mouthwash (IFC), it was not surprising that a superior performance in preventing demineralization was observed with this mouthwash formulation. Furthermore, with the multiple mechanisms of action of CPC, it was not surprising that the mouthwash formulation combining fluoride with only CPC was observed to be the next in rank of effectiveness in preventing caries with 58% reduction of demineralization relative to the control group treatment with de-ionized distilled water (Table 3). It is noteworthy that the possible synergistic effect between fluoride and Isomalt in mouthwash combining fluoride with only Isomalt (IF) was not as pronounced as in IFC, considering that the effectiveness of IF in inhibiting demineralization was comparable to the observed with mouthwash containing only fluoride. Thus, Isomalt showed a better caries preventive effect when combined with CPC than when combined with only fluoride (Figs. 1 and 2; Table 3).

Considering that every tested formulation contains 1100 ppm of fluoride, it was not surprising that all tested mouthwash formulations significantly inhibited caries development, though to a varying percentage (Figs. 1 and 2; Table 3), The ability of different fluoride formulations to prevent dental caries by inhibiting tooth tissue demineralization and retarding the progression of initial caries has long been established with a high level of supporting evidence [6, 42–44]. However, it is pertinent to note that only mouthwash formulations combining fluoride, Isomalt and CPC were significantly more effective in inhibiting demineralization than mouthwash with only fluoride (Figs. 1 and 2). This can be attributed to possible synergistic effect of Isomalt and CPC as stated in the above paragraph. Besides, the caries inhibition action of fluoride is dose-dependent, with the standard concentration (1100–1500 ppm) being unable to provide higher caries prevention in a poor oral hygiene condition as simulated in the present study [45, 46].

The clinical significance of this study, which is a message to the patients receiving isomalt-based mouthwash as a caries control product in clinical practice, is the fact that the presence of isomalt in a fluoride mouthwash enhances the effectiveness of the mouthwash for caries prevention.

However, it is important to mention that one limitation in this study is using de-ionized distilled water as the control mouthwash instead of Phosphate buffered saline (PBS) that is a non-toxic solution. Unlike water, PBS prevents cells from rupturing or shriveling up due to osmosis.

## Conclusion

Within the limit of this study, all the tested mouthrinse formulations inhibited tooth surface demineralization, irrespective of assessment method (SMH or TMR), but mouthrinse formulation combining 1% isomalt, 225 ppm fluoride, and 0.05% cetylpyridinium chloride exhibited the most effectiveness. Thus, mouthrinse formulation containing 1% Isomalt, 225 ppm fluoride, and 0.05% CPC was more effective in preventing caries development in the presence of dental plaque than mouthrinse with only 225 ppm fluoride.

## Data Availability

The data presented in this study are available upon reasonable request from the corresponding author (B.A.).
